# Intracoronary injection of nitroglycerine can prevent unnecessary percutaneous coronary intervention

**DOI:** 10.1186/s12872-022-02823-2

**Published:** 2022-09-18

**Authors:** Amirhossein Nasiri-Partovi, Akbar Shafiee, Reza Rahmani

**Affiliations:** 1 Department of Cardiology, Imam Khomeini Hospital, Tehran University of Medical Sciences, Dr. Gharib st, Keshavarz Blvd, Tehran, 1419733141 Iran; 2 Department of Cardiovascular Research, Tehran Heart Center, Cardiovascular Research Institute, Tehran University of Medical Sciences, Tehran, Iran

**Keywords:** Intracoronary nitroglycerine, Coronary artery disease, Percutaneous coronary intervention

## Abstract

**Background:**

Despite the recommendation of the current guidelines, intracoronary administration of nitroglycerine during coronary angiography is often neglected. We investigated the effect of intra-coronary nitroglycerin on the relief of coronary artery stenosis in the candidates for percutaneous coronary intervention (PCI).

**Methods:**

We included patients with angina pectoris or myocardial infarction who were candidates for PCI. In the coronary angiography, the culprit vessel involved was evaluated, and bolus nitroglycerin at a dose of 25–200 mcg was injected into the affected coronary artery. A significant change in the percentage of coronary artery stenosis was considered a positive response, and these patients were then compared with patients who did not have a substantial change in the percentage of stenosis at the same time. Univariate analysis and then multivariate logistic regression analysis was performed to determine the predictors of response to intracoronary nitroglycerin.

**Results:**

Among 360 patients, 27 (7.5%) responded to nitroglycerine, and 333 (92.5%) were non-responsive. The mean age of patients was 60.2 ± 11.6 years, ranging from 23 to 93 years, and 265 (73.6%) were men. The study groups were not significantly different in the baseline demographic characteristics. The presence of multivessel disease (Odds ratio (OR) = 16.26, 95% confidence interval (CI):2.07–127.6; *P* = 0.008) and stenosis in the left circumflex artery (OR = 3.62, 95% CI: 1.03–12.70; *P* = 0.044) were the independent predictors for nonresponse to nitroglycerine, leading to PCI.

**Conclusion:**

In some cases, especially those without multivessel diseases, intracoronary nitroglycerine administration can efficiently relieve coronary stenosis and prevent unnecessary PCI.

## Introduction

Acute coronary syndrome (ACS) is a common clinical condition in the emergency departments, and patients with elevation of cardiac biomarkers, electrocardiographic changes, or typical clinical symptoms of unstable angina are directed for further evaluation by coronary angiography [1, 2]. Percutaneous coronary intervention (PCI) is currently the mainstay of treatment for symptomatic coronary artery disease [3, 4]. However, this safe procedure is not free of complications, and it can sometimes fail to reach complete revascularization [5, 6]. On the other hand, not all patients with ACS have coronary stenosis, and current evidence shows that up to 30% of them do not require PCI [7]. Among the probable causes, coronary spasm is a frequent mechanism for ACS, causing ischemia at rest.

Coronary artery spasm, a reversible, focal, and severe vasoconstriction, could happen in almost 1.5% of PCI procedures [8, 9]. Coronary artery spasm was observed in 25% of patients who presented with stable angina and almost 49% of those with ACS [7]. Also, it is more probable to occur at the site of a recent myocardial infarction and complicates fixed coronary lesions in up to 60% of acute coronary syndrome cases [10]. Previous studies have shown that intra-coronary injection of nitroglycerin has been effective in relieving stenosis in patients with coronary vasospasm and, in some cases, in reducing atherosclerotic stenosis [11–13]. Moreover, it is noteworthy that many patients with coronary vasospasm are treated invasively as an atherothrombotic coronary event [8]. However, the effect of intra-coronary nitroglycerin administration during angiography has not received much attention in clinical practice yet, and despite the recommendation of the current guidelines, it is often neglected in clinical practice [3, 14, 15]. We believe administering intracoronary nitroglycerins during diagnostic coronary angiography and before PCI may significantly reduce unnecessary invasive coronary revascularization. Therefore, we investigated the effect of intra-coronary nitroglycerin administration on coronary artery stenosis in patients undergoing PCI in our hospital.

## Materials and methods

In this prospective cohort study, we evaluated the effect of intra-coronary nitroglycerin on the relief of coronary artery stenosis in patients undergoing PCI. Participants in this study were selected from PCI candidates referred to Imam Khomeini Hospital between 2018 and 2019. Patients with angina pectoris or myocardial infarction and having significant coronary stenosis (> 70%) were included in the study. Two independent interventional cardiologists reviewed coronary arteriograms. The percentage of stenosis was calculated as follows using the Cardiovascular measurement software (QAngio XA 7.2, MEDIS): 100 × [1- (stenosis diameter/reference diameter)]. In these measurements, the minimum lesion diameter was used to calculate the percentage of stenosis diameter relative to the reference vessel diameter [16].

The research board of the cardiology department and the committee of medical ethics at our center approved the protocol of this study (IR.TUMS.IKHC.REC.1397.252). All participants gave informed consent to take part in the study.

Before enrolment, patients' characteristics, including age, sex, cardiovascular risk factors, and the diagnosis at presentation, including stable angina, unstable angina, non-ST elevation myocardial infarction (NSTEMI), and ST-elevation myocardial infarction (STEMI) were recorded. Risk factors for coronary artery disease included hypercholesterolemia, hypertension, smoking, chronic kidney disease, and diabetes mellitus [17, 18].

In the case of significant stenosis during angiography, nitroglycerin at a dose of 25–200 mcg was injected into the affected coronary arteries as a bolus dose. The dosage was decided individually for every patient based on the type of vessel, its diameter, and hemodynamics of the patient at the discernment of the interventionist. The percentage of stenosis before and after intra-coronary nitroglycerin injection was assessed. Response to nitroglycerine was defined as the reduction of the stenosis below 70% of the lumen. Unresponsive patients to intracoronary nitroglycerine injection with significant coronary stenosis (> 70%) or with moderate coronary stenosis (40–70%) but fractional flow reserve (FFR) less than 0.8 underwent PCI. The planned PCI was suspended in responsive patients to intracoronary nitroglycerine with stenosis below 40% or with moderate coronary stenosis (40–70%) but FFR more than 0.8.

### Statistical analysis

The categorical data were expressed as frequency (percentage) and were compared between the groups using the Chi-square test. Numeric data were shown as mean ± standard deviation and were compared between the group using the student's *t*-test. We performed univariable and multivariable logistic regression analyses to determine the predictors for response to nitroglycerine. For the multivariable regression model, variables with a *P*-value < 0.2 in the univariable model were included. Odds ratios (OR) and 95% confidence intervals (CI) were calculated and reported for every variable. The statistical analysis was performed using SPSS version 24.0 (IBM, USA), and a *P*-value of < 0.05 was considered significant.

## Results

**Fig. 1:**
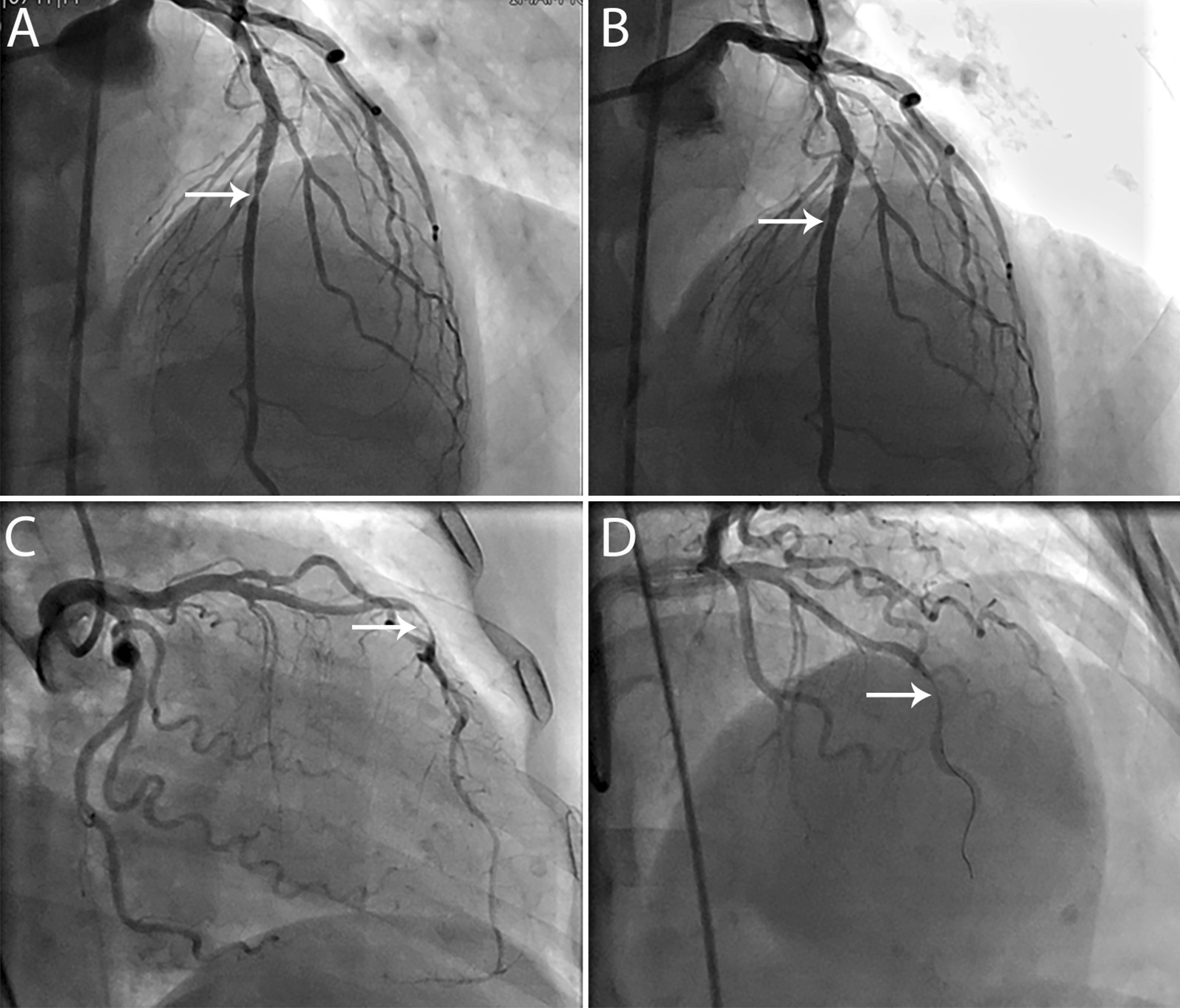
**A** Coronary angiography of a 74 year old man showing stenosis in the mid section of the left anterior descending artery that resolved after intracoronary nitroglycerine injection (**B**). **C** Coronary angiography of a 75 year old woman showing stenosis in the mid section of the left anterior descending artery that did not resolve after administration of intracoronary nitroglycerine (**D**)

In this study, we evaluated the clinical and angiographic data of 366 patients. In total, 27 (7.5%) patients were responsive to intracoronary nitroglycerine (responsive group), and 333 (92.5%) patients were unresponsive (unresponsive group). The mean age of the patients was 60.2 ± 11.6 years, ranging from 23 to 93 years. Men constituted 73.6% of the patients. There was no significant difference between the study groups in the baseline demographic characteristics. However, a history of atherosclerotic cardiovascular disease seemed to be more frequent in the unresponsive group (*P* = 0.081). Multivessel disease was more frequent in the unresponsive group (*P* = 0.002) and the stenosis in the left anterior descending artery (*P* = 0.006). Table 1 summarizes the details of the baseline and angiographic characteristics of the study population and their comparison between the responsive and nonresponsive groups. Response and nonresponse to intracoronary nitroglycerine in two identical patients are depicted in Fig. 1

**Table 1:** Baseline characteristics of the study population, their comparison between the responsive and unresponsive patients to nitroglycerine, and the univariable logistic regression analysis

	Total, n = 360	Responsive (n = 27)	Unresponsive (n = 333)	Odds ratio	95 confidence interval	*P*-value
Age, year (mean ± SD)	60.2 ± 11.6	60.8 ± 10.5	60.2 ± 11.7	0.99	0.96–1.03	0.796
Male sex, n (%)	265 (73.6)	18 (66.7)	247 (74.2)	1.43	0.62–3.31	0.397
Hypertension, n (%)	176 (48.9)	11 (40.7)	165 (49.5)	1.43	0.64–3.17	0.381
Diabetes mellitus, n (%)	106 (29.4)	5 (18.5)	101 (30.3)	1.92	0.71–5.20	0.202
Hyperlipidemia, n (%)	81 (22.5)	4 (14.8)	77 (23.1)	1.73	0.58–5.15	0.325
Smoking, n (%)	117 (32.5)	10 (37.0)	107 (32.1)	0.8	0.36–1.82	0.601
Previous cardiovascular disease, n (%)	138 (38.3)	6 (22.2)	132 (39.6)	2.3	0.90–5.85	0.081
*Diagnosis at presentation, n (%)*						
Stable angina	128 (35.6)	13 (48.1)	115 (34.5)	–	–	0.058
Unstable angina/NSTEMI	83 (23.1)	9 (33.3)	74 (22.2)	0.93	0.38–2.28	0.873
STEMI	149 (41.4)	5 (18.5)	144 (43.2)	3.26	1.13–9.40	0.029
*The involved vessel, n (%)*						
LM stenosis	19 (5.3)	3 (11.1)	16 (4.8)	0.4	0.11–1.48	0.172
LAD stenosis	223 (61.9)	10 (37.0)	213 (64.0)	3.02	1.34–6.80	0.006
LCX stenosis	112 (31.1)	4 (14.8)	108 (32.4)	2.76	0.93–8.17	0.067
RCA stenosis	144 (40.0)	11 (40.7)	133 (39.9)	0.97	0.43–2.15	0.935
Multivessel disease, n (%)	159 (44.2)	1 (3.7)	158 (47.4)	23.47	3.15–174.99	0.002

*LAD* left anterior descending artery; *LCX* left circumflex artery; *LM* left main coronary artery; *NSTEMI* Non-ST elevation myocardial infarction; *RCA* right coronary artery; *SD* standard deviation; *STEMI* ST-elevation myocardial infarction

*P < 0.05 was considered significant

The multivariate analysis showed the presence of multivessel disease (OR = 16.26, 95% CI:2.07–127.6; *P* = 0.008) and stenosis in the left circumflex artery (OR = 3.62, 95% CI: 1.03–12.70; *P* = 0.044) were the independent predictors for nonresponse to nitroglycerine, leading to PCI (Table 2). Nonetheless, presenting with STEMI was a borderline predictor for nonresponse to nitroglycerin (OR: 3.02, 95% CI: 0.98–9.36; *P* = 0.055).

**Table 2:** Predictors of response to intracoronary nitroglycerine based on the multivariable logistic regression analysis

Characteristic	Odds ratio	95% Confidence interval	*P*-value
Previous cardiovascular disease	0.419	0.15, 1.168	0.096
*Diagnosis at presentation*			
Stable angina, n (%)	–	–	0.060
Unstable angina/NSTEMI, n (%)	0.72	0.26–1.99	0.532
STEMI, n (%)	3.02	0.98–9.36	0.055
LM stenosis	0.32	0.07–1.58	0.163
LAD stenosis	2.18	0.87–5.48	0.096
LCX stenosis	3.62	1.03–12.70	0.044
MVD stenosis	16.26	2.07–127.6	0.008

*P < 0.05 was statistically significant

## Discussion

This study investigated the effect of intracoronary nitroglycerin administration on relieving coronary artery stenosis in PCI candidates. All patients had significant coronary stenosis (> 70%) before injection and were candidates for revascularization. Nevertheless, 7.5% of patients responded to it after intracoronary administration of nitroglycerine and did not require PCI. The independent predictors for response to nitroglycerin included multivessel disease and stenosis in the left circumflex artery.

Coronary vasospasm is a reversible, focal, and intense coronary vasoconstriction, and the diagnosis is generally made in people without significant coronary stenosis [8]. As far as the prognosis of patients with coronary artery spasm is excellent [17], diagnosis before performing any coronary procedure is essential. Reactivity of the coronary artery to antispasmodic agents can be a helpful way to diagnose coronary vasospasm and prevent unnecessary stenting and the resulting complications and costs [19, 20]. For this reason, intracoronary administration of nitroglycerine leads to increased coronary flow through spasm relief [21], which was also confirmed in our study.

Several case reports in the literature address the benefit of the administration of intracoronary nitroglycerine in patients who presented with ACS, and this action deferred them from PCI [22–25]. In a case series study by Vishnevsky et al. [14], intracoronary nitroglycerine administration during angiography prior to a scheduled PCI resulted in the resolution of stenosis in all patients. In this study, none of the six patients who responded to intracoronary nitroglycerine had STEMI and were finally treated with calcium channel blockers with or without long-acting nitrates. Vishnevsky's study showed that the possibility of coronary spasm should be considered in patients with coronary artery stenosis on angiography, especially in young people, smokers, or positive history of migraine. However, we did not observe any association between smoking and response to nitroglycerine in the present study. Vishnevsky et al. noted that coronary spasm could be present in severe cases during coronary angiography, even those without symptoms of angina or electrocardiographic changes, although ECG changes were not studied in the present study. Therefore, an intracoronary nitroglycerine bolus in cases of suspected vasospasm will be both diagnostic and therapeutic, as confirmed by our study.

Mohammad et al. also reported 16 patients with left main coronary artery vasospasm who were falsely diagnosed with stenosis and underwent coronary artery bypass graft surgery [26]. To prevent unnecessary bypass surgery, the authors recommended intracoronary nitroglycerin as "a reasonable routine in hemodynamically stable patients with significant LM stenosis."

In a study on patients who presented with myocardial infarction, one patient responded to nitroglycerine and did not require fibrinolytic [27]. Based on the CASPAR study (7), 28% of the patients who presented with ACS did not have any significant lesion after intracoronary injection of nitroglycerine. In another study from the same dataset and after three years of follow-up, none of these patients developed nonfatal myocardial infarction or cardiac death. Nonetheless, 50% continued to have persistent angina, and angiography was repeated in three cases (3.9%). The authors concluded that patients with ACS who show no culprit lesion and suffer coronary spasm have an excellent and significantly better prognosis for survival and coronary events compared with patients with a culprit lesion. In a unique piece of evidence, a patient with multiple narrowings in the coronary artery angiography responded well to intracoronary nitroglycerine and was deferred from PCI [28]. In our study, out of 140 patients with STEMI, 5 (3.5%) responded to nitroglycerine and were treated medically. Considering the massive number of patients who undergo PCI annually, this small percentage can dramatically reduce the costs and complications.

### Study limitations

As mentioned above, the number of patients previously examined was very small. While in this cross-sectional study, 360 patients were studied, of whom 27 were responsive to nitroglycerine, and therefore, previous studies are not comparable to our study in terms of sample size. In addition, these patients were carefully analyzed for details such as risk factors, clinical syndrome, and type of vessel involved. Nonetheless, our study has some shortcomings. First, this was a single-center study performed in a university hospital. Also, we did not follow up with the patients for a more extended period to observe the recurrence of the symptoms or progression to CAD.

## Conclusion

Intracoronary nitroglycerin administration can relieve coronary artery stenosis in the candidates for PCI and prevent unnecessary procedures. The presence of multivessel disease and stenosis in the left circumflex artery were the independent predictors for non-responders. A further large-scale study might clarify the definite predictors of response to intracoronary nitroglycerine.

## Data Availability

The datasets generated and analyzed during the current study are not publicly available due to the policy of our department but are available from the corresponding author on reasonable request.
